# ^225^Actinium-armed antibody targeting CCR8^+^ regulatory T cells synergizes with immunotherapy to promote tumor rejection in syngeneic colorectal cancer models

**DOI:** 10.3389/fimmu.2025.1662216

**Published:** 2025-09-16

**Authors:** Connor Frank, Zhiwen Xiao, Kevin J. H. Allen, Rubin Jiao, Mackenzie E. Malo, Ekaterina Dadachova

**Affiliations:** College of Pharmacy and Nutrition, University of Saskatchewan, Saskatoon, SK, Canada

**Keywords:** ^225^Ac-radioimmunotherapy, immune checkpoint inhibitors, CCR8, regulatory T cells, colorectal cancer

## Abstract

**Background:**

Colorectal cancer (CRC) remains a formidable threat to health worldwide. Immunotherapy with immune checkpoint inhibitors results in only a minority of CRC patients experiencing long-term progression-free survival, at the expense of significant autoimmune toxicity. Development of new therapeutics to “wake up” the immune system to fight CRC is necessary. Here we investigated for the first time radioimmunotherapy (RIT) directed towards CCR8, a marker of tumor-infiltrating immunosuppressive T-regulatory cells (ti-Tregs) as a method to recover anti-tumor immunity followed by immunotherapy in CRC models.

**Methods:**

225Actinium (^225^Ac)-labeled anti-CCR8 antibody and anti-CTLA-4 immunotherapy were used to assess their potential synergistic effects in syngeneic murine CRC models CT26 and MC38. The safety of all treatments was assessed through complete blood counts and blood chemistry. ^225^Ac-anti-CCR8 RIT-treated tumors were analyzed immunohistochemically for FoxP3 and CCR8 expression while mechanistic studies of tumor-infiltrating lymphocytes were done by flow cytometry.

**Results:**

^225^Ac-anti-CCR8 RIT alone demonstrated effectiveness in CRC models but dramatic anti-tumor response was observed when it was combined with anti-CTLA-4 immunotherapy. Immunotherapy alone failed to control tumor growth. Tumor immunohistochemistry post ^225^Ac-anti-CCR8 RIT showed ablation of CCR8^+^ ti-Tregs while flow cytometry analysis revealed CCR8-specific increased influx of effector CD8^+^ T cells, M1 macrophages and NK cells in comparison with ^225^Ac-control antibody.

**Conclusions:**

These data demonstrate a synergistic effect of anti-aCCR8 RIT with immunotherapy through enhancement of adaptive and innate anti-tumor responses. Further investigation of anti-CCR8 RIT as a potential cancer-agnostic agent and its combinations with other immunotherapy agents such as anti-PD-1, LAG3 or TIGIT is warranted.

## Introduction

The advent of cancer immunotherapy has been a significant advancement in the treatment of many malignancies with particularly successful examples being melanoma and bladder cancer (1, 2). However, certain malignancies, such as gastro-esophageal and colorectal cancer, fail to respond to immune checkpoint inhibitor (ICI) therapy such as Keytruda™ (anti-programmed cell death protein 1, anti-PD-1) or Yervoy™ (anti-cytotoxic T-lymphocyte-associated protein 4, anti-CTLA-4) (3, 4). The mechanisms driving this failure to respond to ICI are still being investigated, however, a few key components driving resistance have been elucidated. A crucial component of the ICI response failure is the inability to initiate anti-tumor responses due to inadequate activation of anti-tumor CD8^+^ T cell function within the tumor microenvironment (TME) (5). Secretion of immunosuppressive cytokines such as transforming growth factor-beta and recruitment of specialized regulatory cells including CD4^+^FoxP3^+^ T regulatory cells (Tregs) and myeloid derived suppressor cells which suppress anti-tumor immune response have been correlated with poor outcomes in patients (6–8).

There is a need to counteract such immunosuppression to recover anti-tumor immune responses in the context of ICI. Recent work has identified the surface C-C motif chemokine receptor 8 (CCR8) as a highly restricted marker for the tumor-infiltrating Tregs (ti-Tregs) within human and murine tumors (9). While blockading CCR8 on ti-Tregs in tumors in murine cancer models did not improve therapeutic outcomes of anti-PD-1 therapy, depletion of CCR8^+^ ti-Tregs with natural killer (NK) cells in antibody dependent cellular cytotoxicity (ADCC)-capable mice demonstrated synergistic tumor regression with anti-PD-1 ICI (10). However, cellular therapy is a complex and very expensive type of treatment. An alternative way of targeted elimination of CCR8^+^ ti-Tregs is radioimmunotherapy (RIT). Targeted radionuclide therapy is currently experiencing growth with recent approval of Lutathera™ and Pluvicto™ for treating neuroendocrine tumors and metastatic prostate cancer, respectively (11, 12). Radioimmunotherapy (RIT) is a subset of targeted radionuclide therapy. RIT targets radiation at the molecular level utilizing radiolabeled monoclonal antibodies (mAbs) that bind to overexpressed or uniquely cancer specific antigens located either on the cancer cell membrane. RIT can precisely deliver highly cytotoxic radionuclides to localized or systemic cancer foci, while reducing potential side effects (13). There is also a lot of interest towards combining targeted radionuclide therapy with immune checkpoint inhibitors with promising results emanating from the experimental studies and clinical trials (14). To the best of our knowledge, RIT has not been utilized before for elimination of ti-Tregs from the tumors. We hypothesized that highly selective and potent irradiation of CCR8-positive ti-Tregs with alpha particles emitted by Actinium-225 (^225^Ac)-labeled mAb to CCR8 (anti-CCR8 mAb) will selectively eliminate ti-Tregs in the tumors and enhance immunotherapy with ICI. In this proof of principle study, we utilized MC38 and CT26 syngeneic colorectal cancer models in immunocompetent mice to assess immune modulation and outcomes after RIT and ICI immunotherapy. Here, we report the results of synergistic combination of ^225^Ac-anti-CCR8 RIT with ICI and its effects on TME.

## Materials and methods

### Reagents and radionuclides

Purified rat IgG2b anti-mouse CCR8 (Clone: SA214G2, Cat# 150302) was purchased from BioLegend (San Diego, CA, USA). Isotype matched rat IgG2b k control antibody (Clone: RTK4530, Cat#400602) was also purchased from BioLegend. Mouse IgG2b anti-mouse CTLA-4 (Clone: 9D9, Cat#BE0164) was purchased from BioXCell (Lebanon, NH, USA). ^225^Ac was purchased from Oak Ridge National Laboratory (Oak Ridge, TN, USA). ^111^In was purchased from BWXT (Cambridge, ON, Canada). All other reagents are supplied from ThermoFisher unless otherwise stated.

### Conjugation and radiolabeling

Anti-CCR8 and control antibodies were conjugated with 10M excess of bifunctional chelating agent p-SCN-Bn-DOTA (Macrocyclics, Plano, TX, USA) as in (15) and radiolabeled as described in **Supplementary Methods**.

### Cell lines

Mycoplasma free murine colorectal adenocarcinoma cell lines MC38 and CT26.WT (CRL-2638) were received from ATCC (Gaithersburg, MD, USA). MC38 cells were maintained in DMEM high glucose + L-glutamine (Cytiva, Cat#SH30022.01) supplemented with 10% fetal bovine serum (Cytiva, Cat#SH30070.03). CT26.WT cells were maintained in RPMI 1640 + L-glutamine supplemented with 10% fetal bovine serum. Cells were grown in a humidified incubator maintained at 37°C + 5% CO_2_.

### Animal studies

Animal studies were reviewed and authorized by the University of Saskatchewan Animal Care Committee and were performed under institutional animal use protocol number 20170006. Female 9 week old Balb/c (Strain 028) or C57Bl/6 (Strain 027) mice were obtained from Charles River Laboratories (Senneville, QC, Canada) and acclimatized in the animal facility for one week before being used in the experiments. Mice were housed in groups of 5 per cage in a temperature controlled and pathogen free housing with ad libitum access to water and food during all studies. For MC38 and CT26 tumor models, cells were harvested and resuspended in a 1:1 mixture of chilled complete cell medium and cold Cultrex Reduced Growth Factor Basement Membrane Extract (R&D Systems, Cat# 3433-005-01) at the specified concentration of cells. Balb/c and C57BL/6 mice were inoculated subcutaneously on the right flank with 100 µL of solution DMEM+cells:Cultrex containing either 2.5x10^5^ CT26 cells or 5.0x10^5^ MC38 cells, respectively. Animals were monitored daily for tumor growth and imaging or therapeutic studies were initiated at ~100 mm^3^ tumor volumes. Mice were randomized according to their tumor size, animals with larger or smaller tumors were excluded. During therapy studies measurement of tumor volume was done in different order on the days of measurement to avoid bias. CF and ZX were blinded when measuring the tumor volume and performing statistical analysis at the end of the experiments. During therapy studies the approved animal protocol allowed for tumor volume of up to 4,000 mm^3^ which constituted the endpoint of the study. If the tumor was interfering with the animal’s movement or feeding, become necrotic or if an animal lost more ≥20% their bodyweight before the tumor reached 4,000 mm^3^ – that animal was immediately humanely euthanized.

### Micro single photon emission computed tomography/computed tomography imaging and biodistribution

MC38 and CT26 ~100 mm^3^ tumor bearing female C57Bl/6 and Balb/c mice, respectively, were injected intravenously (IV) with 7.4 MBq ^111^In-aCCR8 or ^111^In-control mAb. Mice were imaged on a MILabs Vector4 microSPECT/CT camera (Utrecht, Netherlands) using a XUHS-M collimator at 24, 48 and 72 hours post radiolabeled antibodies administration. SPECT images were acquired at 245 keV and 171 keV ^111^In gamma emissions. Images were processed with MILabs software (v8.00RC6) using 0.4 mm voxel grid with 10 iterations and 10 subsets, then filtered via gaussian smoothing 3D FWHM (2 mm X, 2 mm Y, 2 mm Z) using pMOD software v3.910 (pMOD Technologies, Zurich, Switzerland). For visual representation of accumulation MIP (maximum intensity projection) images were utilized. At 144 hours post-injection of radiolabeled mAb mice were humanely sacrificed for the biodistribution, their tumor, liver, pancreas, small intestine and spleen were collected, weighted, and their radioactivity measured in a gamma counter. The percentage of injected doses per gram organ (%ID/g) as well as time activity curves were calculated and plotted.

### ^225^Ac-anti-CCR8 mAb monotherapy

When CT26 and MC38 tumors reached ~ 100 mm^3^ volume, the Balb/c and C57Bl/6 mice, respectively, were randomized according to their tumor size into groups of 5 animals each and treated with: 1 - unlabeled anti-CCR8 mAb; 2 – 7.4 kBq/200 ng ^225^Ac-anti-CCR8 mAb; 3 – 14.8 kBq/400 ng ^225^Ac-anti-CCR8 mAb; 4 - left untreated. Mice in each group were observed for the minimum of 20 days and their tumor growth and body weight measured every 2 days. Tumor growth was measured with electronic calipers (volume = length × width^2^/2). Any animal with a necrotic tumor was humanely sacrificed. At the conclusion of the study the tumors were analyzed by immunohistochemistry (IHC) for presence of FoxP3+ and CCR8+ cells as described below.

### ^225^Ac-anti-CCR8 mAb combination with anti-CTLA-4 immunotherapy

In these experiments we investigated if the combination of CCR8-targeting RIT with anti-CTLA-4 antibody immunotherapy could produce an additive or synergistic effect on the tumor progression. CT26 and MC38 tumors in mice were initiated as above and when tumors reach ~ 100 mm^3^ - the tumor-bearing mice were randomized into groups of 5 animals each according to the tumor size and treated with: 1 - 7.4 kBq/200 ng ^225^Ac-anti-CCR8 and a loading dose (10 mg/kg, 200 µg) of anti-CTLA-4 immunotherapy; 2 - 7.4 kBq/200 ng ^225^Ac-anti-CCR8; 3 - a loading dose (10 mg/kg, 200 µg) of CTLA-4 immunotherapy; 4 – left untreated. Subsequently, on Days 2 and 4, the second and third rounds of anti-CTLA-4 immunotherapy (5 mg/kg, 100 µg) injections were administered to mice in groups 1 and 3. The mice were observed for their tumor size, survival and body weight for up to 42 days.

### Evaluation of hematologic and systemic toxicity of monotherapy and combination treatments

CT26 and MC38 tumor bearing mice were randomized as above and treated with various doses of ^225^Ac-anti-CCR8 mAb monotherapy and a combination of ^225^Ac-anti-CCR8 mAb and anti-CTLA-4 immunotherapy as described above. At the completion of observation period the blood was collected and analyzed for white blood cells (WBC), red blood cells (RBC), platelet, liver toxicity (aspartate aminotransferase (AST) and alanine transaminase (ALT)) and kidney toxicity (creatinine and blood urea nitrogen (BUN)).

### Tumor infiltrating lymphocyte isolation and flow cytometry

CT26 and MC38 tumor bearing mice were randomized as above and treated with 7.4 kBq of ^225^Ac-anti-CCR8 mAb and the tumors were harvested on Days 3 and 7 post treatment for flow cytometric analysis. Tumor infiltrating lymphocytes were isolated as described in **Supplementary Methods**. Freshly isolated tumor and spleen lymphocytes were washed twice in sterile PBS (Cytiva, Cat# SH30256.02) and analyzed by flow cytometry as described in **Supplementary Methods**.

### Immunohistochemistry

The goal of the qualitative IHC studies was to detect the presence of Tregs and CCR8+ Tregs in the untreated tumors as well as their presence or absence in the tumors post treatment with ^225^Ac-anti-CCR8 antibody at the end of experimental observation of treated animals. MC38 and CT26 tumors were fixed in 4% paraformaldehyde, then stored in 70% alcohol, and embedded in paraffin blocks. Immunohistochemistry was performed as described in **Supplementary Methods**.

### Statistical analysis

Power analysis of RIT studies was done with PASS version 11 (NCSS, Inc.) using simulations of different tumor volumes based on pilot data and conservative assumptions regarding the groups treated with the radiolabeled antibodies. All simulations showed power of at least 83% with only five animals per group because of the large differences between treated and untreated animals. Thus, 5 mice per group were utilized in the RIT and mechanistic studies. All mice which participated in the experiments were included into the analysis. Data was analyzed via two-way ANOVA with Tukey’s multiple comparisons using Prism software (Graphpad, CA, USA). A two-sided p value <0.05 was considered statistically significant for all studies.

## Results

### CCR8^+^ ti-Tregs are present in colorectal tumors as confirmed by flow cytometry, IHC and *in vivo* microSPECT/CT imaging

**Figure 1:**
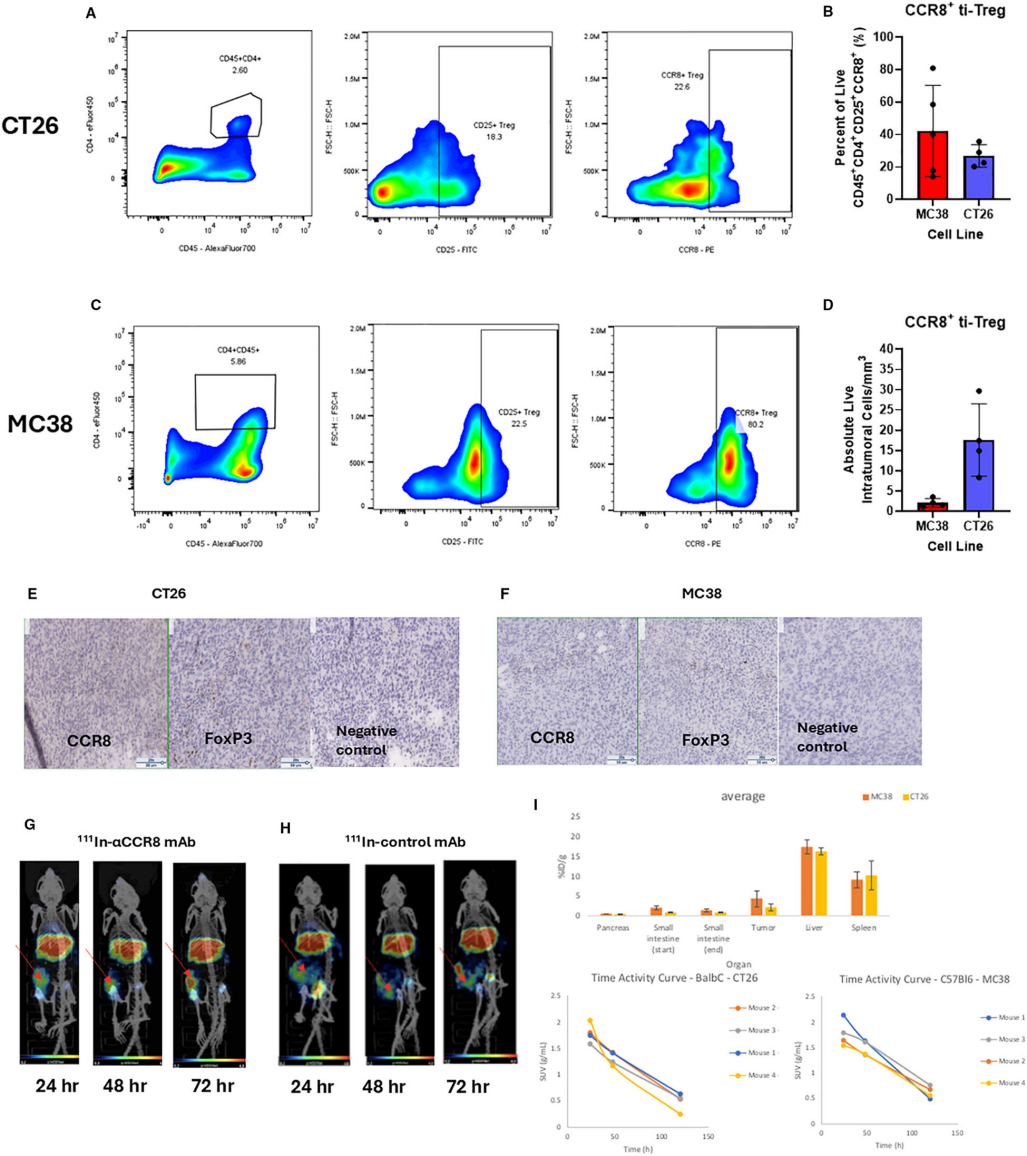
Flow cytometry, IHC and microSPECT/CT analysis of tumor infiltrating Treg (ti-Treg). **(A, C)** Representative gating plots of CD45^+^CD4^+^CD25^+^CCR8^+^ ti-Tregs from **(A)** CT26 murine colorectal adenocarcinoma and **(C)** MC38 colorectal adenocarcinoma. **(B)** Percentage of Tregs expressing CCR8 (data presented as n=5 MC38, n=4 CT26 ± SD). **(D)** Number of live intratumoral CCR8 + ti-Treg cells per mm^3^ of digested tumor. **(E, F)** Immunohistochemistry of CCR8 and FoxP3 markers in **(E)** CT26 and **(F)** MC38 tumors. Brown staining shows CCR8 and FoxP3 positive cells. The slides were stained with either anti-FoxP3 mAb (Clone: FJK-16s), or anti-CCR8 mAb (Clone: SA214G2), or with rat IgG2b (Clone RTK4530) as an isotype negative control. **(G, H)** MicroSPECT/CT images of CT26 tumor-bearing Balb/c mice administered 7.4 kBq of **(G)** ^111^In-anti-CCR8 mAb or **(H)** ^111^In-control mAb at 24, 48 and 72 hr post administration of radiolabeled antibody. Red arrows point to the tumors in the right flank. **(I)** biodistribution conducted at the termination of the imaging experiment at 144 hr post administration shows percentage of injected doses per gram organ (%ID/g) in tumor, liver, pancreas, small intestine and spleen for MC38 and CT26 tumor-bearing mice (upper panel); and time activity curves showing standardized uptake values (SUV’s) (lower panels).

To evaluate the presence of CCR8+ ti-Tregs in mouse tumor models, we performed intratumoral lymphocyte flow cytometry on tumors, (**Supplementary Figure S1**). We established the presence of CD45^+^CD4^+^CD25^+^CCR8^+^ ti-Tregs in MC38 and CT26 colorectal tumor models that constituted more than 40% of all tumors infiltrating Tregs (**Figures 1A–D**). In contrast, spleen housed comparatively small percentage of CCR8^+^ T-regulatory cells (**Supplementary Figure S2**). Flow cytometry findings of ti-Tregs in the tumors were confirmed by IHC staining of CCR8^+^ ti-Tregs (**Figures 1E, F**) while no staining with the isotype control antibody was observed. Finally, *in vivo* presence of CCR8^+^ ti-Tregs was established by administering ^111^In-labeled anti-CCR8 and control mAbs to tumor bearing mice and imaging them with microSPECT/CT. We found that the uptake of ^111^In-labeled anti-CCR8 mAb in the tumors (**Figure 1G**) was much more intense in comparison with the uptake of the ^111^In-labeled control antibody after 24, 48 and 72 hrs (**Figure 1H**), the images from control group mice showed that radioactivity was effusing from the tumors after 48 and 72 hrs which is consistent with enhanced permeability (EPR) effect. The confirmation of CCR8^+^ ti-Treg within tumors validated the pursuit of therapeutic assessment of anti-CCR8 RIT. The biodistribution conducted at the termination of the imaging experiment at 144 hr post administration showed low uptake of the ^111^In-labeled anti-CCR8 mAb in the pancreas (around 1%ID/g) and small intestine (around 2% ID/g) for both Balb/c and C57Bl/6 mice. Uptake of ^111^In-labeled anti-CCR8 mAb in the liver and spleen in both mouse strains as well as time activity curves showing standardized uptake values (SUV’s) were typical for murine mAbs at late timepoints post administration (**Figure 1I**).

### RIT with ^225^Ac-labeled anti-CCR8 mAb resulted in delayed tumor growth and elimination of CCR8^+^ ti-Tregs

**Figure 2:**
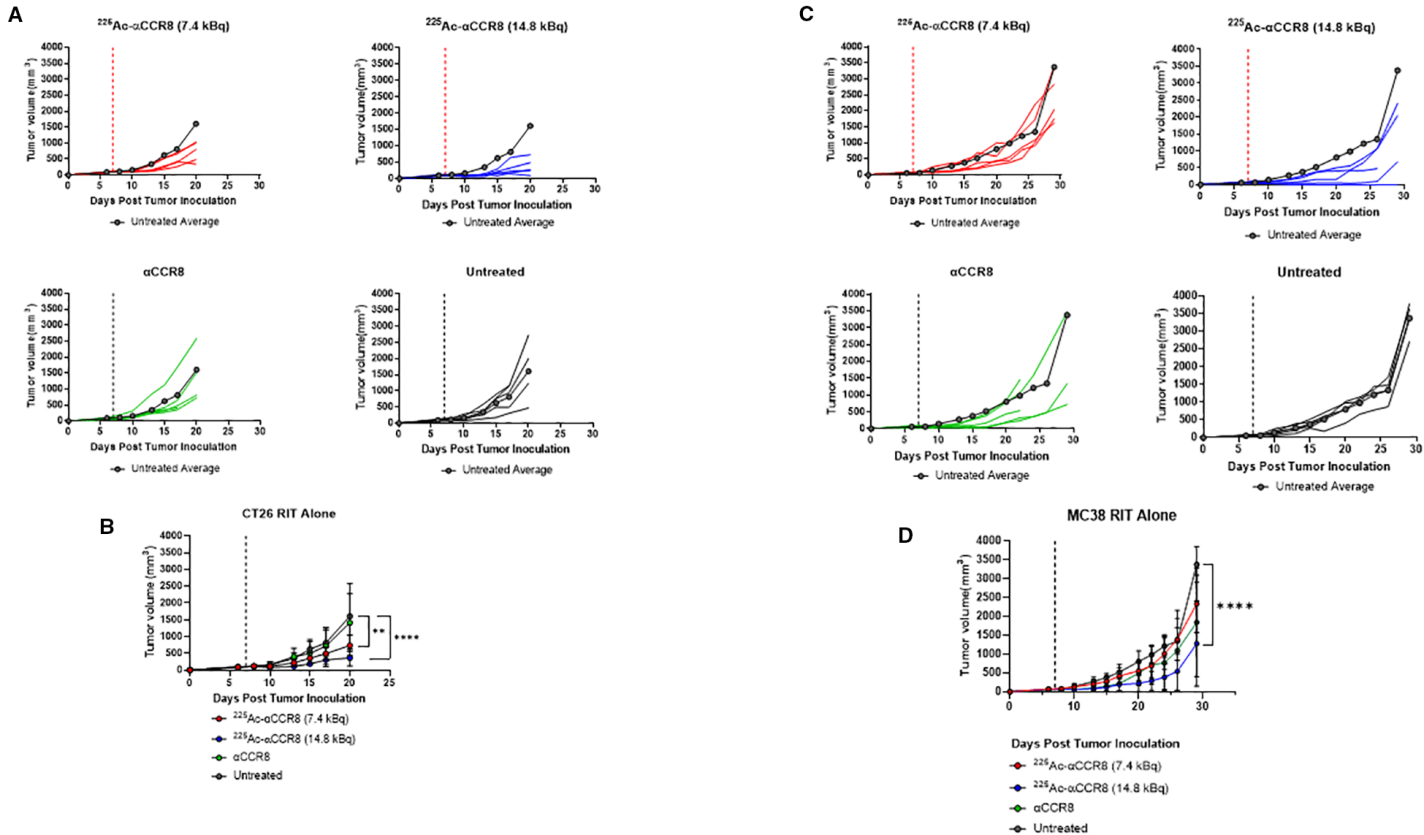
CCR8 targeted RIT monotherapy in CT26 and MC38 colorectal adenocarcinoma. **(A)** Individual tumor growth plots of CT26 bearing Balb/c mice treated with 7.4 kBq ^225^Ac-anti-CCR8, 14.8 kBq ^225^Ac-anti-CCR8, unlabeled anti-CCR8 mAb or left untreated (n=5/group). Dashed lines represent day of treatment. **(B)** Average tumor growth of CT26 bearing Balb/c mice treatment groups (data presented as n=5/group ± SD). Untreated average plotted in grey for reference. p<0.0332 (*), <0.0021 (**), <0.0002 (***) and <0.0001 (****). **(C)** Individual tumor growth plots of MC38 bearing C57Bl/6 mice treated with 7.4 kBq ^225^Ac-anti-CCR8, 14.8 kBq ^225^Ac-anti-CCR8, unlabeled anti-CCR8 mAb or left untreated (n=5/group). **(D)** Average tumor growth of MC38 in treatment groups (data presented as n=5/group ± SD).

To investigate the effects of ^225^Ac-anti-CCR8 RIT on the tumor growth as well as on the numbers of CCR8^+^ ti-Tregs, we performed RIT of CT26 (**Figures 2A, B**) and MC38 (**Figures 2C, D**) tumor bearing mice with ^225^Ac-anti-CCR8 mAb. Both CT26 and MC38 grew aggressively in untreated mice, with MC38 tumors demonstrating faster tumor growth than CT26 ones. Unlabeled anti-CCR8 mAb had no effect on tumor growth in both models. In contrast, when tumor bearing mice were treated with ^225^Ac-anti-CCR8 mAb - there was statistically significant slowing down of CT26 and MC38 tumor growth with 14.8 kBq ^225^Ac-anti-CCR8 mAb and with 7.4 kBq ^225^Ac-anti-CCR8 mAb - of CT26 tumors. To evaluate possible decrease of CCR8+ ti-Tregs within the tumor post-RIT, the tumors were harvested on the day of experiment termination for analysis by IHC. No CCR8^+^ ti-Tregs were detected at both doses for both tumors demonstrating dramatic effect of ^225^Ac-anti-CCR8 RIT (**Supplementary Figure S3**).

### ^225^Ac-anti-CCR8 RIT synergizes with anti-CTLA-4 immunotherapy in CT26 and MC38 colorectal cancer models

**Figure 3:**
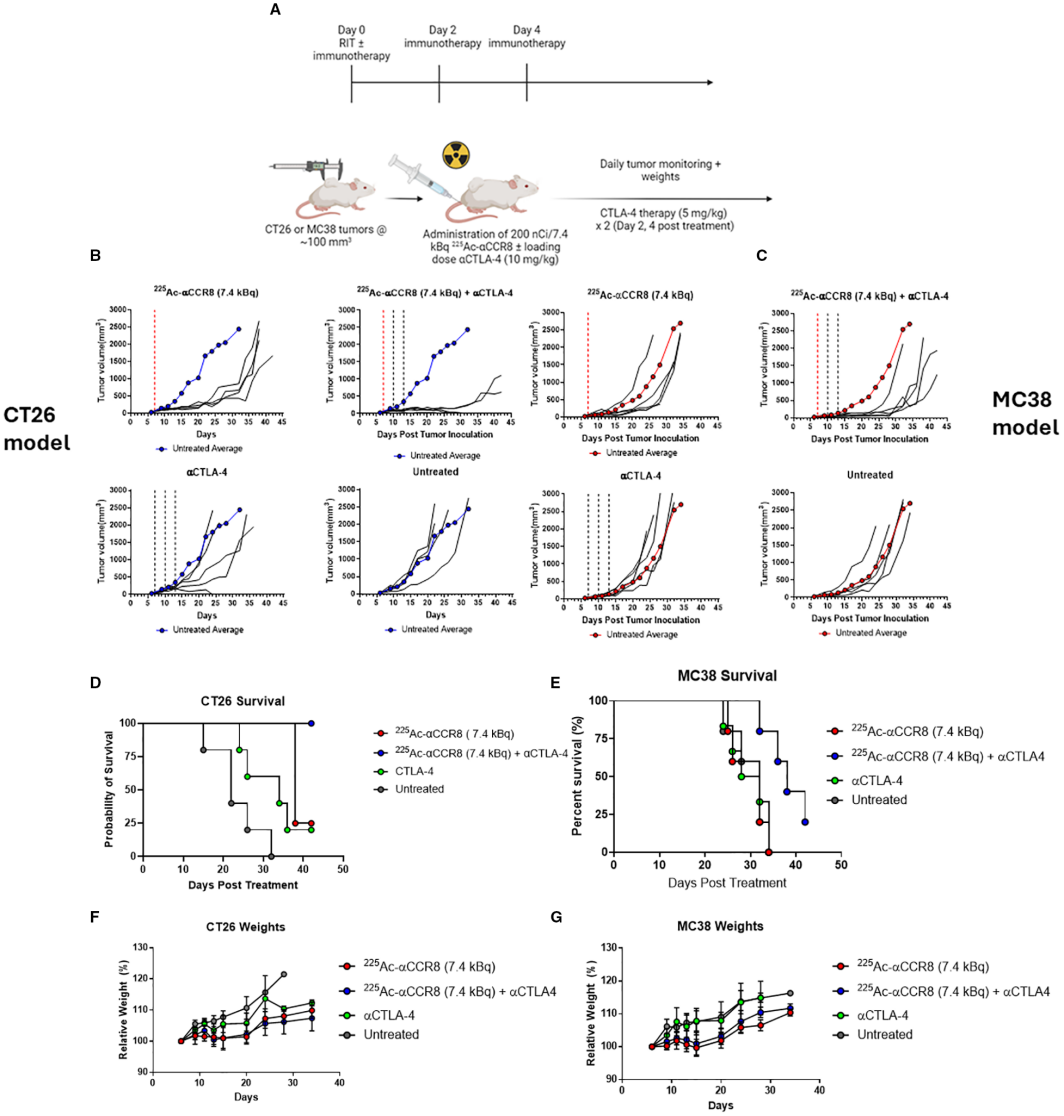
^225^Ac-anti-CCR8 RIT synergizes with anti-CTLA-4 mAb in CT26 and MC38 models and this combination significantly prolongs survival. **(A)** Diagram of study schedules, created with BioRender. **(B)** CT26 tumor bearing Balb/C mice treated with combination of αCCR8 RIT ± aCTLA-4 immunotherapy (n=5/group). Untreated average presented in blue for reference. **(C)** MC38 tumor bearing C57Bl/6 mice with combination of αCCR8 RIT ± αCTLA-4 immunotherapy (n=5/group). Each black line represents individual mouse tumor growth over study period. Untreated average presented in red for reference. Red dotted line indicates RIT administration, black lines – immunotherapy administration; Combination therapy significantly prolonged survival in **(D)** CT26 and **(E)** MC38 tumor bearing mice and did not cause weight loss. **(F)** Weighs of CT26 tumor bearing mice, **(G)** weight of MC38 tumour bearing mice over treatment period.

We investigated the efficacy of combination therapy in both CT26 and MC38 tumor bearing mouse models. As preceding experiments with ^225^Ac-anti-CCR8 mAb revealed elimination of CCR8^+^ ti-Tregs with either 7.4 or 14.8 kBq - we chose a 7.4 kBq ^225^Ac-anti-CCR8 to avoid masking any synergistic effects and to minimize possible toxicity. When tumor size reached ~100 mm^3^ volume, mice received 7.4 kBq of ^225^Ac-anti-CCR8 and a loading dose (10 mg/kg, 200 µg) of CTLA-4 immunotherapy. Subsequently, on Days 2 and 4, the second and third rounds of CTLA-4 immunotherapy (5 mg/kg, 100 µg) injections were administered (**Figure 3A**). **Figures 3B, C** present the individual and average tumor volumes for CT26 and MC38 tumor bearing mice, respectively. Both RIT alone and combination groups displayed decrease in the tumor growth compared with the untreated group and the immunotherapy alone group. Interestingly, 60% of CT26 tumor bearing mice treated with combination therapy showed tumor disappearance and the remaining tumors experienced significant growth inhibition, resulting in smaller tumor sizes compared to any other treatment group during the same period. Combination treatment groups in both models exhibited a significant difference (CT26: p<0.002; MC38 p<0.05) in tumor size compared to the RIT alone groups. The emergence of this significant difference indicates that combination therapy demonstrated a synergistic effect between RIT and CTLA-4 immunotherapy.

### Combination of ^225^Ac-anti-CCR8 RIT with anti-CTLA-4 immunotherapy is well tolerated and significantly prolongs survival

Combination of ^225^Ac-anti-CCR8 and anti-CTLA-4 therapy significantly prolonged survival compared to anti-CTLA-4 therapy alone in both models (CT26: p=0.0018, MC38: p=0.0112) (**Figures 3D, E**). In fact, in CT26 model 100% of mice in combination treatment group survived till the experiment termination with 25% survival in immunotherapy alone group. In more aggressive MC38 model 25% survival was recorded in combination therapy group at the point of experiment termination, while no mice survived in immunotherapy alone group. The combination treatment was well tolerated with mice gaining weight at the same rate as all other groups (**Figures 3F, G**) and with hematological, renal and hepatic parameters staying within their respective normal ranges (**Supplementary Figures S4**, **S5**).

### Anti-CCR8 RIT resulted in expansion of CD4^+^CD8^+^ double positive T cells and 4-1BB^+^ effector CD8^+^ T cells

**Figure 4:**
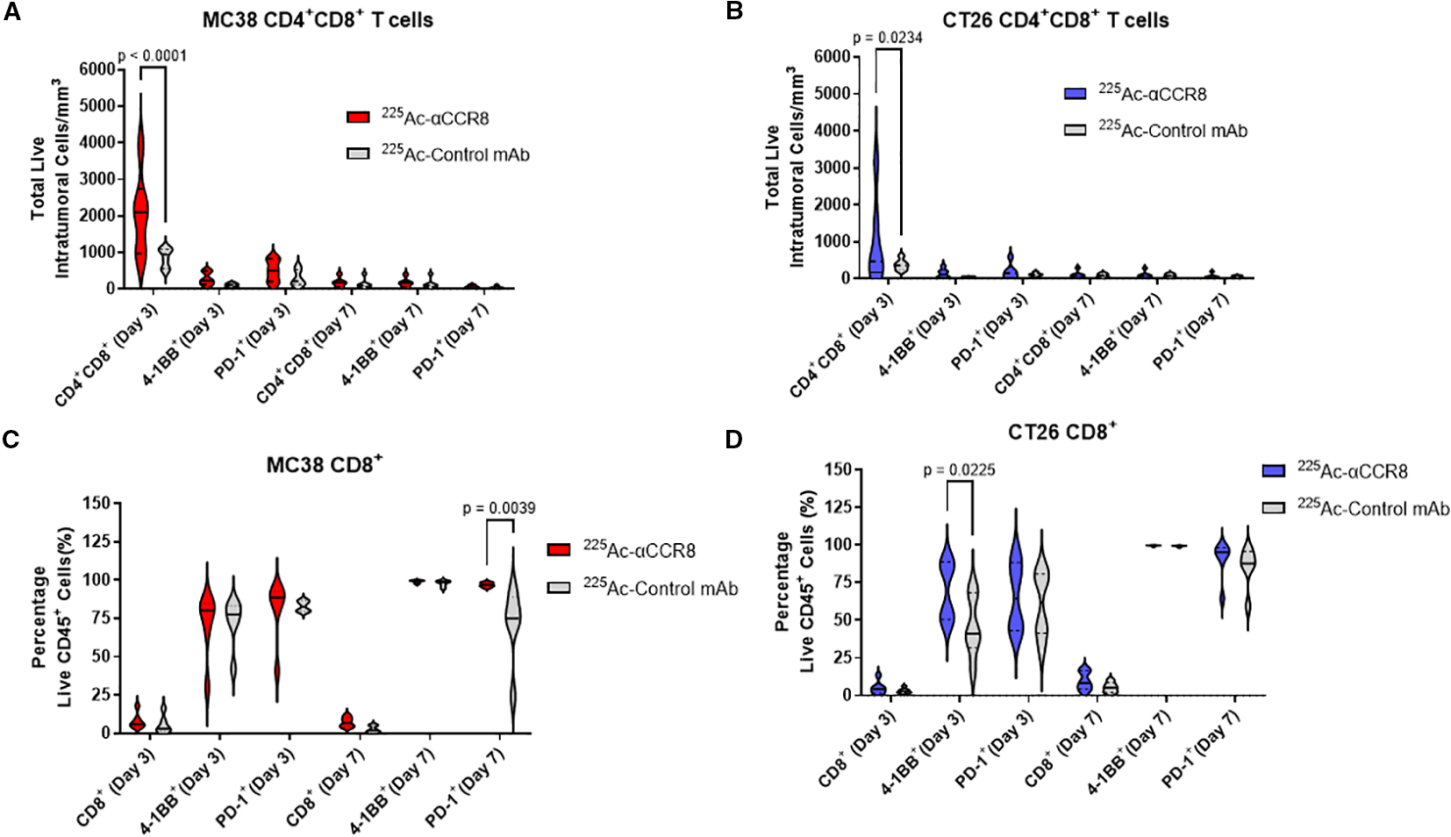
^225^Ac-anti-CCR8 RIT promotes an anti-tumor CD8 T cell responses. **(A, B)** CD4+CD8+ cells in MC38 **(A)** and CT26 **(B)** tumors on Days 3 and 7 post treatment with 7.4 kBq ^225^Ac-anti-CCR8 RIT; **(C, D)** CD8+ cells in MC38 **(C)** and CT26 **(D)** tumors on Days 3 and 7 post treatment with ^225^Ac-anti-CCR8 RIT.

To elucidate the mechanism of anti-CCR8 RIT, flow cytometry panels for detection of T-lymphocytes, macrophages, and NK cells were developed and applied to CT26 and MC38 tumors from mice on Days 3 and 7 post administration of 7.4 kBq ^225^Ac-anti-CCR8 or ^225^Ac-isotype matched control mAbs. A dramatic expansion of CD4^+^CD8^+^ double positive (DP) T lymphocytes was observed on Day 3 post administration of ^225^Ac-anti-CCR8 in both cancer models (CT26: p=0.0234, MC38: p<0.0001). (**Figures 4A, B**). In ^225^Ac-anti-CCR8 treated mice, early expansion on Day 3 of 4-1BB^+^CD8^+^ T cells was observed for CT26 tumors (p=0.0225) (**Figure 4D**). This effect was not observed for MC38 tumors (**Figure 4C**). Interestingly, PD-1 expression on CD8^+^ T cells in MC38 tumors was significantly decreased by Day 7 in control treated tumors (p=0.0039). We have also complied t-SNE (t-distributed stochastic neighbor embedding) maps of CT26 and MC38 tumors treated with ^225^Ac-anti-CCR8 and ^225^Ac-control mAbs on Days 3 and 7 post RIT administration clearly showing CCR8+ Tregs, PD-1+CD8+ T cells and CD45+PD1+ T cells as well as the difference between the numbers of CCR8+ Tregs in ^225^Ac-anti-CCR8 and ^225^Ac-control mAbs groups starting to manifest on Day 7 (**Supplementary Figures S6**, **S7**). We observed large lymphocyte populations (in particular 4-1BB and PD-1, correlating with TME activation) in ^225^Ac-anti-CCR8 mAb treated tumors.

### Anti-CCR8 RIT resulted in expansion of anti-tumor macrophages

**Figure 5:**
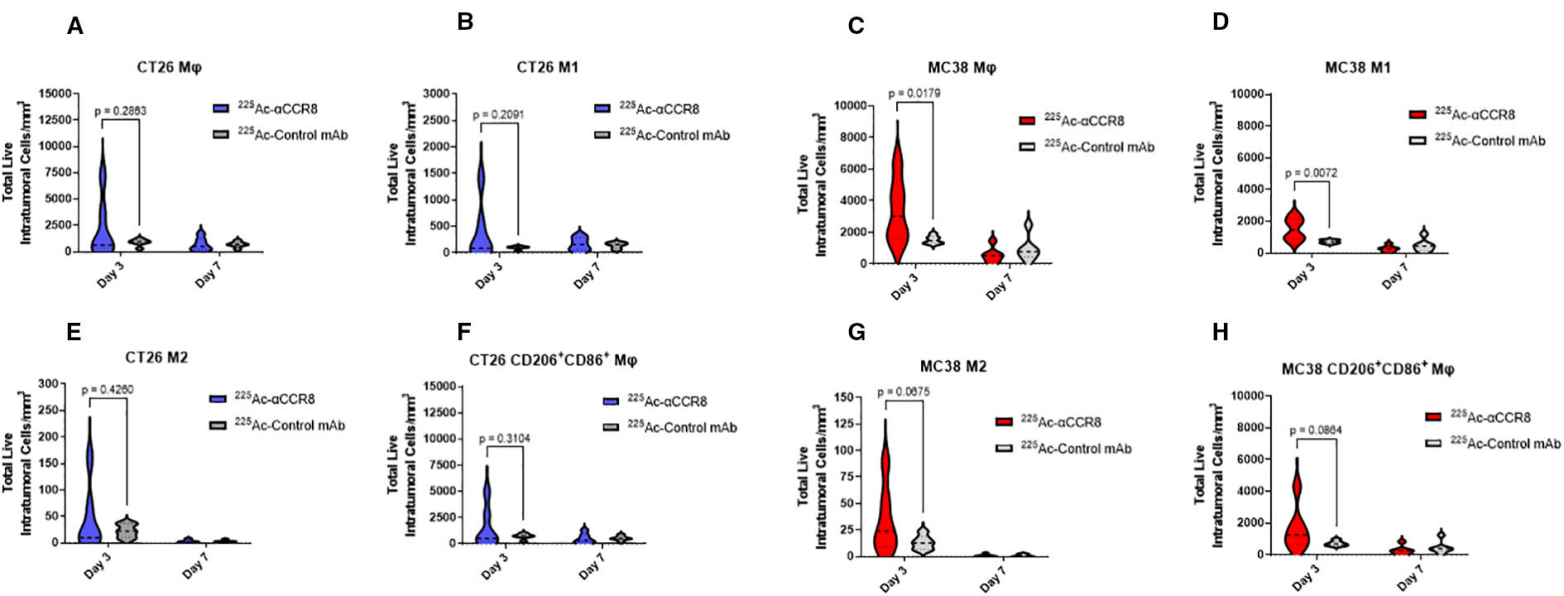
^225^Ac-anti-CCR8 RIT expands anti-tumor macrophage phenotypes on Days 3 and 7 post treatment. **(A, C)** total number of macrophages in CT26 and MC38 tumors, respectively; **(B, D)** M1 macrophages in CT26 and MC38 tumors, respectively; **(E, G)** M2 macrophages in CT26 and MC38 tumors, respectively; **(F, H)** CD86^+^CD206^+^ macrophages in CT26 and MC38 tumors, respectively.

A trend of initial expansion of total number of macrophages and of anti-tumor M1 macrophages was observed on Day 3 post treatment of tumor bearing mice with 7.4 kBq ^225^Ac-anti-CCR8 in both CT26 (**Figures 5A, B**) and MC38 (**Figures 5C, D**) tumors. M2 macrophages, characterized by the expression of CD206, were observed in low amounts in both models (**Figures 5E, G**). ^225^Ac-anti-CCR8 treatment resulted in modest expansion of M2 macrophages (CT26: p=0.2460, MC38: p=0.0675), however, this cells population was low and represented <5% of total Mφ numbers within the tumor. On the contrary, the population of M1/M2-like macrophages expressing both CD86 and CD206 (**Figures 5F, H**) was several folds higher and showed trend towards increase in both CT26 and MC38 treated tumors by Day 3 (CT26: p= 0.3104, MC38: p=0.0675). This effect was diminished by Day 7 post treatment in both tumors.

### Anti-CCR8 RIT resulted in expansion of NK cells

Finally, an expansion of NK cells was observed in both cell lines on Day 3 post treatment with ^225^Ac-anti-CCR8 RIT (**Supplementary Figures S8A, B**) (MC38: p=0.0186; CT26: p=0.1008). In particular, expansion of NKp46^+^ NK cells was observed on Day 3 in MC38 tumors (p=0.0135) (**Supplementary Figure S8C**). Taken together, these observations of expansion of several immune cells in TME post ^225^Ac-anti-CCR8 RIT align with the decreased tumor growth observed after ^225^Ac-anti-CCR8 and CTLA-4 immunotherapy combination treatment.

## Discussion

Cancer immunotherapy has become an extremely clinically relevant modality for the treatment of certain cancer types. The utility of converting immunologically “cold” tumors into “hot” tumors is an intense field of research in the cancer immunology field. Methods to restore and revitalize anti-tumor immune responses may present a possible solution to ICI acquired resistance in many cancer types (16). Previously combinations of immunotherapy with external beam irradiation of the tumors which is not specific for any type of tumor cells or TME have been described (17). In the field of targeted radionuclide therapy we and others reported expansion of certain immune cells in TME post radionuclide therapies directed against cancer cells (18, 19). To the best of our knowledge, here we report the first use of radionuclide therapy directed against CCR8 positive ti-Tregs, a crucial component of TME.

We have chosen immunotherapy-resistant murine colorectal tumors CT26 and MC38 as experimental system. Both tumors responded in a dose dependent manner to anti-CCR8 RIT alone via so called “bystander effect” on the tumor cells initiated by alpha-radiation emitted by ^225^Ac (20). As an immunotherapy we used anti-CTLA4 antibody as anti-CTLA4 antibodies bind to CTLA4 thus releasing the brakes on the cytotoxic T cells in the tumors. Anti-CTLA-4 antibodies, especially in combination with other immunotherapies like anti-PD-1/PD-L1, have shown promising results in colorectal cancers with MSI-high (MSI-H) (21). Importantly, a novel anti-CTLA-4 antibody called botensilimab is being investigated for its potential to treat refractory colorectal cancers, including those with microsatellite stability (MSS) (22). And while no response to anti-CTLA-4 monotherapy was observed - anti-CCR8 RIT and anti-CTLA-4 immunotherapy combination produced significant synergistic effect on the tumors retardation and animals overall survival (**Figure 3**).

Mechanism of action experiments that designed to find an immunological explanation of RIT and immunotherapy revealed expansion of CD4^+^CD8^+^ double positive T cells, anti-tumor M1 macrophages, 4-1BB^+^ effector CD8^+^T cells and NK cells after administration of ^225^Ac-anti-CCR8 RIT (**Figures 4**, **5**, **S6–S8**). Importantly, this action was CCR8-specific as was demonstrated by using the ^225^Ac-control mAb in mechanistic experiments. It has been previously established that tumor inducible acquisition of CD4 and CD8 expression represents a polyfunctional subset of T cells within tumors that have undergone T cell receptor (TCR) stimulation. Double positive T cells express higher levels of PD-1, granzyme B and Ki-67, demonstrating an activated and proliferative phenotype (23). Consistently, our results align with previously published data showing that in this case, anti-CCR8 RIT therapy likely disinhibits T cells from ti-Treg immunosuppression, thus allowing expansion of double 21 positive cells in both cell lines (**Figure 4**). Increased 4-1BB^+^CD8^+^ T cells in this context are indicative of increased TCR activation and priming of anti-tumor CD8^+^ cells. Increase in PD-1+ cells on Day 7 in control antibody treated MC38 tumors may represent decreased TCR stimulation in control treated animals as PD-1 is rapidly upregulated and sustained following TCR stimulation (24). Currently, it is hypothesized that the double expression of CD86^+^CD206^+^ represents a transitional M1/M2 macrophage phenotype. Upregulation of CD86 is associated with loss of CD206 (25). In this case, double expression of CD86^+^CD206^+^ aligns with the observed expansion of M1 macrophages with a large population of M2 to M1 transitional macrophages. NK cell mediated tumor killing is often executed following the activation of receptors from the natural cytotoxicity receptor family, including NKp46 (26). These results suggest RIT is causing the remodeling of TME. Taken together, the observed synergy is based on the combination of three mechanisms: 1) RIT off target killing of tumor cells via “bystander” effect (20); 2) RIT directly killing CCR8+ Tregs which allows anti-CTLA4 immunotherapy to become more effective; 3) RIT re-modelling TME by inviting NK cells and anti-tumor macrophages into TME.

Encouragingly, RIT alone and its combination and immunotherapy did not produce any non-transient hematological or systemic toxicity for up to 40 days post-treatment. It has been shown in previous studies by us and other groups that the doses of ^225^Ac-labeled mAbs equal or below 18.5 kBq per mouse do not cause acute hematological or systemic toxicity besides transient drop in WBC, platelet and body weight at nadir after administration of radiolabeled mAbs (15, 27–29). Determination of potential long term toxicity of ^225^Ac-anti-CCR8 mAb including non-tumor tissues which are known to host Tregs (skin, gut) and CCR8+ Tregs (spleen) should be conducted during the pre-clinical phase by treating healthy mice with the escalating doses of ^225^Ac-anti-CCR8 mAb, observing them for up to 12 months and performing histological analysis of organs for signs of radiation damage.

Most radiopharmaceuticals in preclinical and clinical investigation directly target tumor antigens or receptors present on the cancer cells. We present the evidence that targeting the components of TME, in this case ti-Tregs, is as a potentially cancer-agnostic application of RIT in oncology. These results demonstrate the flexibility of RIT in the context of multiple cancer types and highlights the opportunity for new targets to be investigated in oncology, autoimmune and other diseases where targeted depletion of a cell type would be advantageous. Finally, we highlight the observation that even a small cell population can be of tremendous importance to disease progression. The observation that CCR8^+^ ti-Tregs represent <2% of all CD4^+^ cells within tumors highlights their immense immunosuppressive capability and ability to enhance immune evasion. The importance of multiple immunosuppressive subsets including myeloid derived suppressive cells (MDSC’s) and certain macrophage subsets is recognized. These subsets currently are challenging therapeutically from a RIT perspective due to the increased radiation resistance of macrophage cells, which can withstand up to 10 Gy of absorbed radiation (30). There is also the potential for these subsets to compensate for the lack of immunosuppression from ti-Tregs after targeted depletion. Further investigation into agents selective for depletion of immunosuppressive subsets within the TME is an opportunity to develop cancer-agnostic immunotherapy enhancers.

Colorectal cancers are classified as either microsatellite stable (MSS) or microsatellite instability-high (MSI-H) based on their DNA stability. While immune checkpoint inhibitors like nivolumab and pembrolizumab have revolutionized treatment for MSI-H colorectal cancer, MSS tumors are generally resistant to these immunotherapies because they have fewer mutations, which means fewer neoantigens are present (31). For this reason MSS tumors are often described as “immune-cold” because they have a low density of immune cells within TME. Thus, modifying TME in MSS tumors with RIT targeting CCR8+ Tregs could provide a novel strategy to improve the outcomes for patients with MSS tumors.

CCR8 expression by Tregs in the tumors could potentially serve as prognostic indicator for patients as it is possible to label anti-CCR8 antibodies with imaging radionuclides and select candidates for the combination therapy. In this regard, the study of a different set of immune markers - CK18 and GDF5 - in oral squamous cell carcinoma demonstrated how the expression levels of epithelial and differentiation markers provide prognostic insight and reflect the underlying immune and differentiation status of tumors (32).

This proof of principle study has some limitations: 1) murine models may not fully replicate human immune responses; 2) immunological memory and persistence of immune response post-anti-CCR8 RIT has not been investigated. We hypothesize that combination of immune checkpoint inhibitors and targeted ti-Treg therapy would promote long lasting immunological memory through generation of antigen specific CD8^+^ memory cells. Further studies are planned using humanized models or ex vivo human CRC tissues as well as to investigate potential memory effects of ti-Treg depletion and if this enhancement of tumor rejection can be imprinted on the immune system long-term to reject recurrence of tumors.

Lastly, in addition to the colorectal cancer, CCR8+ Tregs presence has been demonstrated in such major types of cancer as breast, hepatocellular carcinoma, non-small cell lung cancer and metastatic melanoma (9, 10, 33–38). Thus, this immunotherapy enhancement platform could likely be expanded to enhance effector T cell targeted immunotherapies such as anti-PD-1, anti-LAG3 or TIGIT to enhance CD8^+^ responses further than what was observed with anti-CTLA-4.

## Conclusion

These results demonstrate that an anti-CCR8 RIT is a promising avenue for the enhancement of a range of immunotherapy agents in a cancer-agnostic fashion. We believe that further investigation in different syngeneic tumor models and immunotherapy agents using anti-CCR8 RIT is warranted.

## Data Availability

The original contributions presented in the study are included in the article/**Supplementary Material**. Further inquiries can be directed to the corresponding author.
