# Supplementary material for: 225Actinium-armed antibody targeting CCR8+ regulatory T cells synergizes with immunotherapy to promote tumor rejection in syngeneic colorectal cancer models
Source: Front Immunol. 2025 Sep 16;16:1662216. doi: 10.3389/fimmu.2025.1662216 (PMC12479553; doi:10.3389/fimmu.2025.1662216)
Supplement: Supplementary file 1 [file DataSheet1.pdf]

Supplementary Data for the manuscript: ^{225}Actinium-armed antibody targeting CCR8^{+} regulatory T cells synergizes with immunotherapy to promote tumor rejection in syngeneic colorectal cancer models

Connor Frank †, Zhiwen Xiao †, Kevin J.H Allen, Rubin Jiao, Mackenzie Malo, Ekaterina Dadachova*

College of Pharmacy and Nutrition, University of Saskatchewan, Saskatoon, SK S7N 5A8, Canada

*Corresponding author: Ekaterina Dadachova: College of Pharmacy & Nutrition, University of Saskatchewan, Saskatoon, S7N 5E5, SK, Canada, Telephone (306) 966-5163; E-mail: ekaterina.dadachova@usask.ca

Supplementary Methods:

Conjugation and radiolabeling

Anti-CCR8 and control antibodies were conjugated with 10M excess of bifunctional chelating agent p-SCN-Bn-DOTA (Macrocyclics, Plano, TX, USA) as in (15). ^{225}Ac or ^{111}In were reacted with conjugated antibody with a specific activity of 37 kBq/µg or 370 kBq/µg, respectively. Labeling was performed at 37°C with shaking for 1 hour and was subsequently quenched with 2 µL of 50 mM DTPA or EDTA for ^{225}Ac and ^{111}In, respectively. Radiochemical yields were measured via radio-iTLC (instant thin layer chromatography) 10 cm strips using 0.15M ammonium acetate mobile phase. iTLC strips were counted on a Wizard 2470 gamma counter (PerkinElmer, Waltham, MA, USA). Radiochemical yield was determined via the following equation:

$$Yield = \frac{(CPM\ iTLC\ bottom)}{(CPM\ iTLC\ bottom)+(CPM\ iTLC\ Top)} * 100$$

The radiolabeling yields for both ^{225}Ac- and ^{111}In-labeled mAbs exceeded 95% and, thus, radiolabeled mAbs did not require purification. The association of radioactivity with the mAb molecule was also confirmed by size exclusion HPLC performed on Agilent HPLC system equipped with Bioscan flowthrough radiation detector.

Tumor infiltrating lymphocyte isolation and flow cytometry

CT26 and MC38 tumor bearing mice were randomized as above and treated with 7.4 kBq of ^{225}Ac-anti-CCR8 mAb and the tumors were harvested on Days 3 and 7 post treatment for flow cytometric analysis. Tumors were manually minced into ~2 mm pieces with a scalpel and transferred into a gentleMACS™ C tube (Miltenyi Biotec, Cat#130- 093-237). Tumor mouse dissociation kit (Miltenyi Biotec, Cat#130-096-730) was used as per manufacturer's guidelines

with minor modification. Reduction of enzyme R by 20% was done to increase tumor infiltrating leukocyte recovery and viability. After tumor digestion, a single cell tumor suspension was obtained and subjected to Ficoll-Paque PREMIUM 1.084 g/mL (Cytiva, Cat#GE17-5446-02) isolation. Briefly, single cell tumor suspension was brought up to 25 mL with DMEM containing phenol red (Cytiva, Cat#SH30022.01) and was transferred into a 50 mL conical tube. 10 mL of Ficoll-Paque PREMIUM was gently pipetted using a slow speed auto-pipettor underneath the tumor mixture. A clear separation of the tumor digestion mixture and Ficoll-Paque was observed prior to centrifugation at 900 x g with 2 acceleration and 0 deceleration settings for 30 minutes at 21°C. After centrifugation, the interface layer containing the tumor infiltrating lymphocytes between the Ficoll and Tumor digestion mixture was collected, washed and processed for flow cytometry analysis as described below.

Freshly isolated tumor lymphocytes were washed twice in sterile PBS (Cytiva, Cat# SH30256.02). Cells were stained using 1:1000 PBS diluted Fixable Viability Dye eFluor™ 506 (Invitrogen, Cat#65-0866-14) in PBS for 10 minutes at 4°C protected from light. Cells were then washed three times using FACS buffer (PBS + 2% FBS + 0.02% Sodium Azide). Cells were then incubated with 1:100 diluted purified anti-mouse CD16/CD32 (Clone: 93, Invitrogen, Cat#14-0161-82) for 10 minutes on ice. Cells were washed three times using FACS buffer and stained for 30 minutes at 4°C with one of the following three panels : 1) T cell panel containing anti-mouse CD4-eFluor 450 (Invitrogen, Clone:GK1.5), anti-mouse PD-1-Brilliant Violet 605 (BioLegend, Clone:29F.1A12), anti-mouse CD25-AlexaFluor 488 (BioLegend, Clone:PC61), anti-mouse CD8-PerCPCy5.5 (Clone:53-6.7), anti-mouse CCR8-PE (Clone: SA214G2), anti-mouse CD137/4-1BB-APC (Clone:17B5) and anti-mouse CD45-AlexaFluor700 (Clone:30-F11); 2) Macrophage panel

containing anti-mouse CD11b-eFluor 450 (Clone:M1/70), anti-mouse CD206/MMR-AlexaFluor 8 488 (Clone:MR6F3), anti-mouse F4/80-PE (Clone:BM8), anti-mouse CD86-APC (Clone: GL-1) and anti-CD45-AlexaFluor 700 (Clone: 30-F11). 3) NK panel containing anti-mouse CD3-FITC (Clone:17A2), anti-mouse NK1.1-PE (Clone:PK136), anti-mouse CD49b-APC (Clone:HMα2) and anti-mouse CD335-APC-Fire750 (Clone:29A1.4). All antibody stainings were performed at a 1:100 dilution in FACS buffer. Cells were washed three times, resuspended in fresh FACS buffer and analyzed using a Beckman Coulter CytoFlex. Data was processed and analyzed using FlowJo software (Version 10.4.).

For spleen tissue Treg staining, spleens were mechanically dissociated using a 0.7 µm cell strainer and resuspended in PBS. Spleen cells were then stained with 1:1000 PBS diluted LIVE/DEAD™ Fixable Near IR (780) dye (Invitrogen, Cat#L34993) for 10 minutes at 4°C. Cells were washed 3x in FACS buffer and incubated with 1:100 FACS buffer diluted purified anti-mouse CD16/CD32 (Clone: 93, Invitrogen, Cat#14-0161-82) for 10 minutes on ice. Cells were washed 3x in FACS buffer and surface stained 1:100 diluted anti-mouse CD4-eFluor 450 (Invitrogen, Clone: GK1.5), anti-mouse CD3-Brilliant Violet 605 (BioLegend, Clone: 17A2) and anti-mouse CCR8-PE (BioLegend, Clone: SA214G2) for 30 minutes at 4°C. Cells were then fixed and permeabilized using eBioscience™ FoxP3/Transcription Factor Staining Buffer Set (Invitrogen, Cat#00-5523-00) as per manufacturers recommendations. Cells were intracellularly stained with anti-mouse FoxP3-APC (Invitrogen, Clone: FJK-16s) for 30 minutes at 4°C. Cells were washed with FACS buffer and analyzed as previously described.

Immunohistochemistry

MC38 and CT26 tumors were fixed in 4% paraformaldehyde, then stored in 70% alcohol, and embedded in paraffin blocks. Samples needed for the experiment were selected and sectioned at 5-10μm using a microtome, heated at 60°C for 1 h, deparaffinized with xylene, and dehydrated in fractions with different concentrations of alcohol. The tumor tissues were stained with hematoxylin. Endogenous peroxidase activity was quenched with 3% hydrogen peroxide in methanol. Heat-induced epitope retrieval was performed in Tris/EDTA, pH=9 buffer containing 10% glycerol for 13 min. The slides were incubated at 4°C overnight using a dilution of 1:250 anti-FoxP3 mAb (Clone: FJK-16s, Cat#14-5773-82) and 1:500 anti-CCR8 mAb (Clone: SA214G2, Cat# 150302). Using 3,3'-diaminobenzidine tetrachloride (DAB) (Dako Canada Inc., Mississauga, ON) as the developing agent and hematoxylin counterstain, primary antibodies were detected with the HRP- 24 labeled polymer detection reagents (En-Vision + System, Dako Canada Inc., Mississauga, ON). Rat IgG2b, κ Isotype Control Antibody (Clone RTK4530) was used as an isotype negative control. Data was acquired using an Aperio Microscope.

Supplementary Figures:

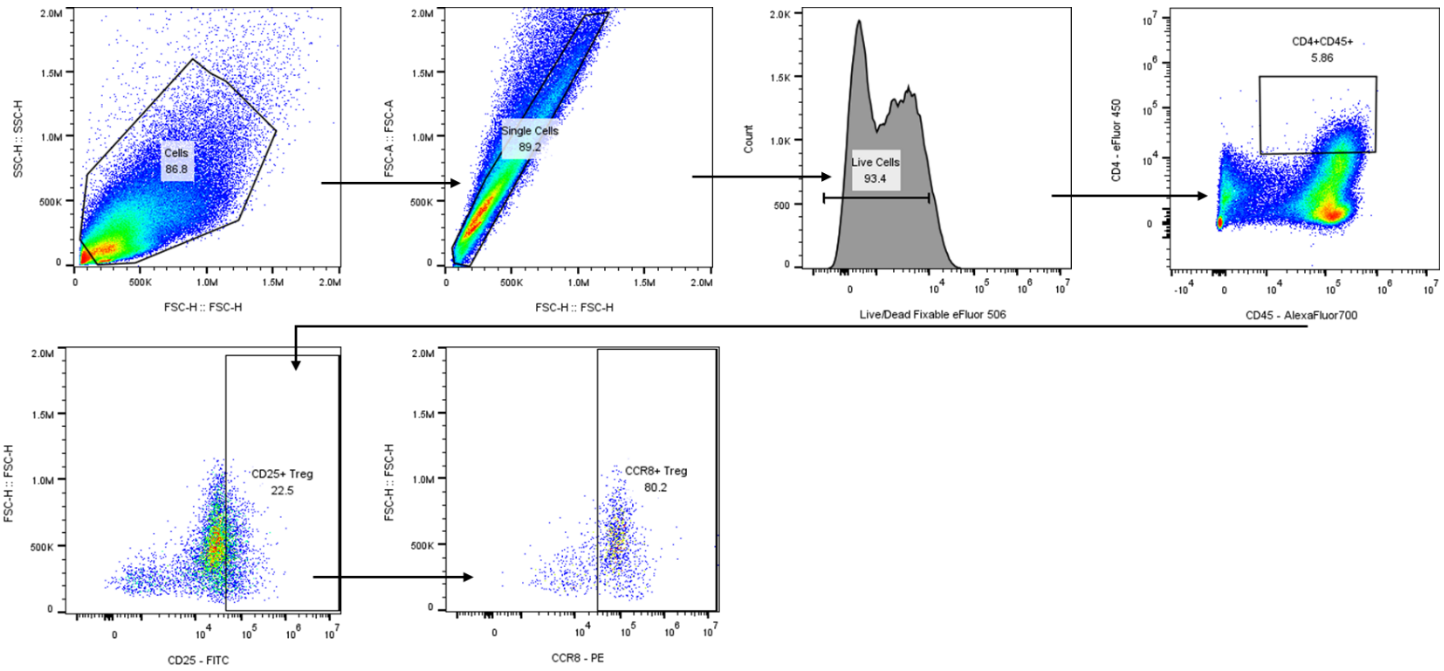

Supplementary Fig. S1 Gating strategy for CCR8+ isolated ti-Tregs from tumor samples. Gating strategy depicted for ti-Tregs isolated from digested CT26 mouse colorectal adenocarcinoma cells.

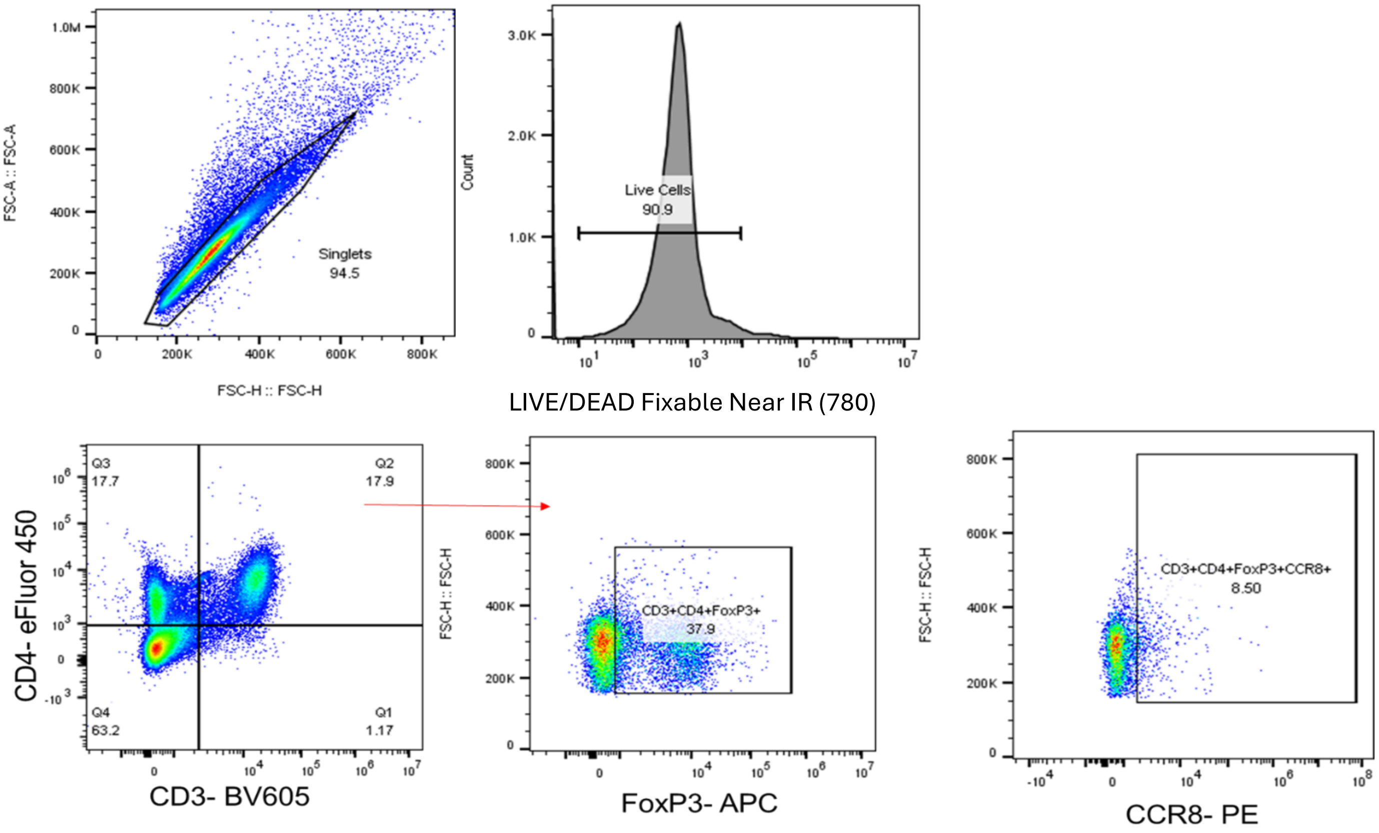

Supplementary Fig. S2 Splenic Tregs express CCR8 at low levels. Gating strategy depicted for splenic Tregs isolated from a Balb/C mouse defined as CD4$^+$CD3$^+$FoxP3$^+$.

CT26 tumors

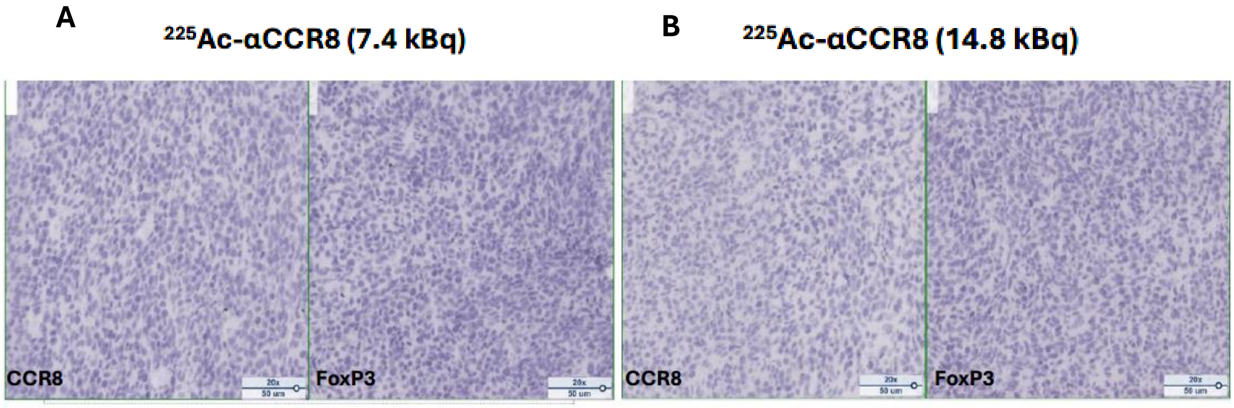

MC38 tumors

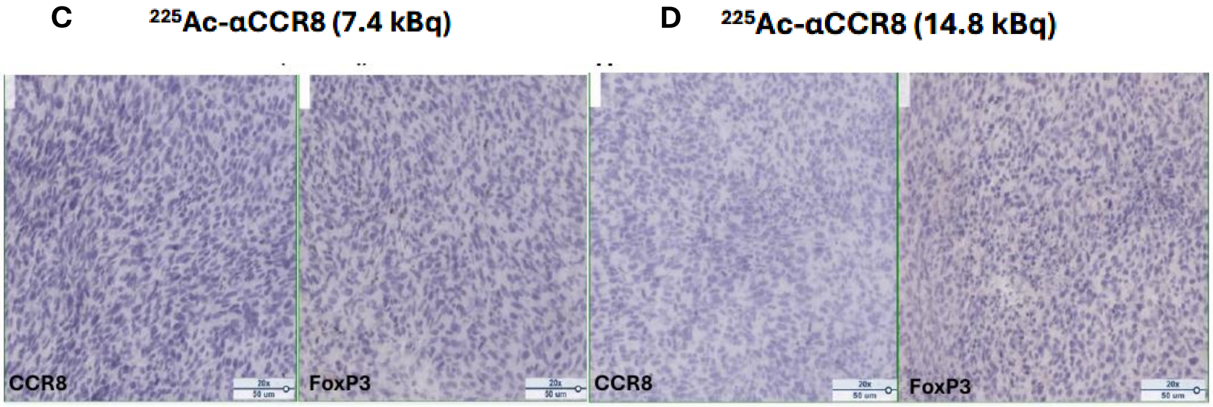

Supplementary Fig. S3 Immunohistochemistry of CT26 (A,B) and MC38 (C,D) tumors from mice treated with 7.4 kBq ^{225}Ac-anti-CCR8 mAb (A,C) or 14.8 kBq ^{225}Ac-anti-CCR8 (B,D). "CCR8" marked slides show staining for CCR8, "FoxP3" marked slides show staining for FoxP3. No staining for both CCR8 and FoxP3 was detected post treatment with ^{225}Ac-anti-CCR8 mAb.

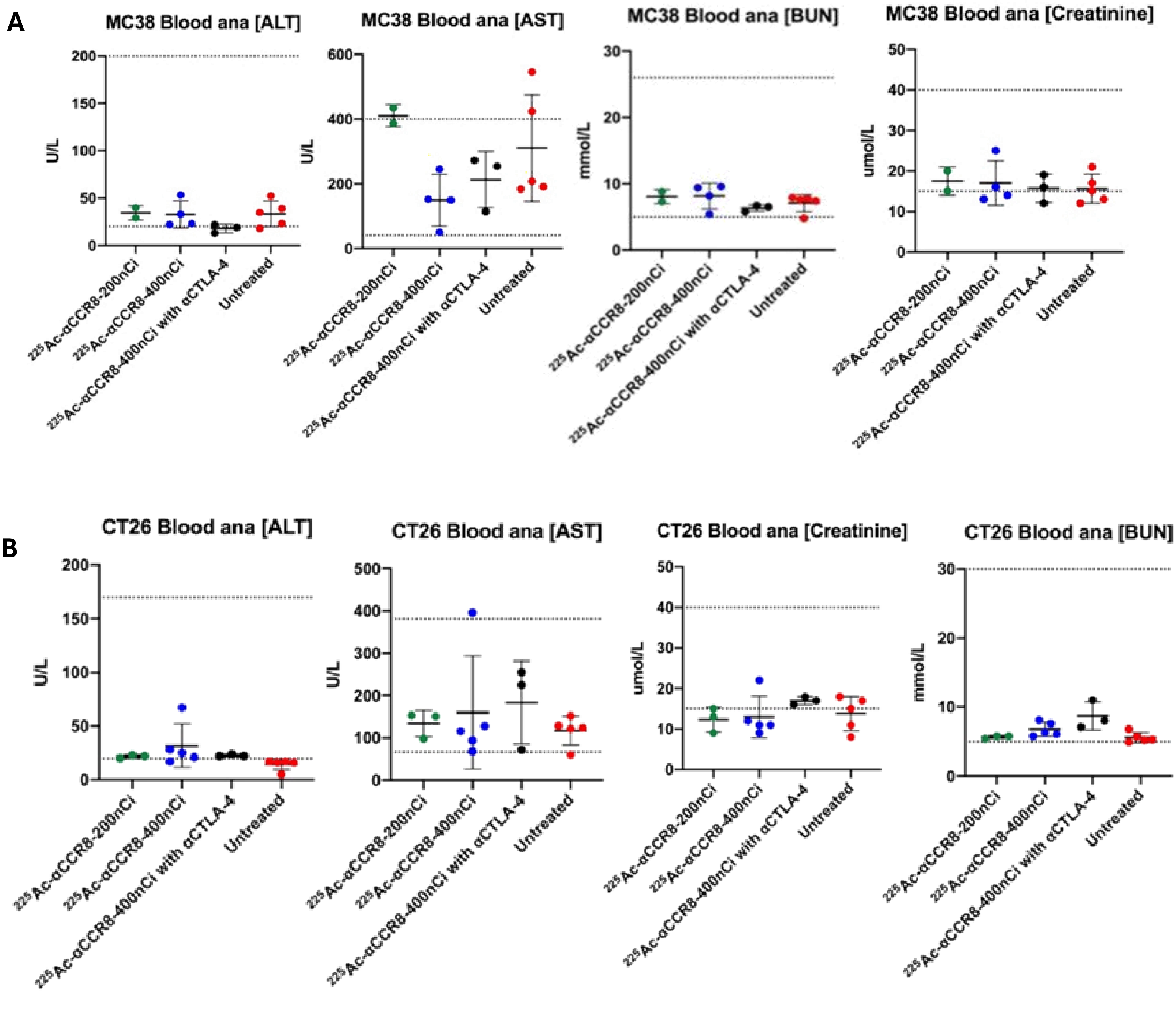

Supplementary Fig. S4 ^{225}Ac-anti-CCR8 combination therapy does not induce hepatic or acute kidney injury. Blood chemistry from MC38 (A) and CT26 (B) tumor bearing mice treated with 7.4 kBq, 14.8 kBq; 14.8 kBq + anti-CTLA-4 immunotherapy; or untreated (data presented as n=3-4/group ± SD). Hashed lines represented normal value ranges for mice provided by manufacturer. Alanine aminotransferase (ALT) and aspartate aminotransferase (AST) presented as enzyme units/litre (U/L). Creatinine presented as micromole/L (µmol/L) and blood urea nitrogen (BUN) presented as micromole/L (mmol/L).

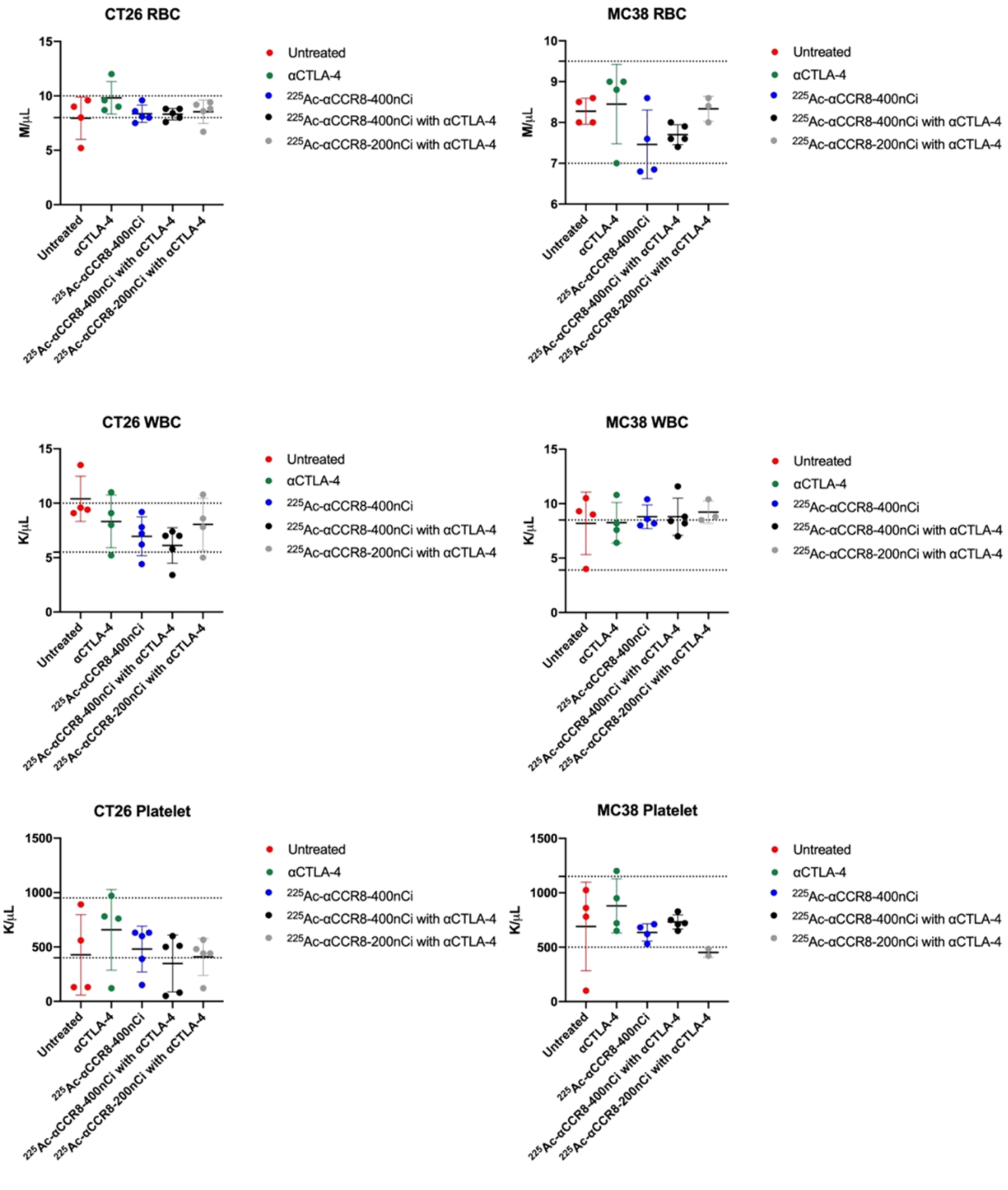

Supplementary Fig. S5 ^{225}Ac-anti-CCR8 does not induce hematological damage in mouse colorectal cancer models. Blood counts from CT26 (left plates) and MC38 (right plates) tumor bearing mice treated with ^{225}Ac-anti-CCR8 at 7.4 kBq, 14.8 kBq, 7.4 kBq + anti-CTLA-4; 14.8 kBq + anti-CTLA-4 immunotherapy; or left untreated (data presented as n=3-4/group ± SD).

RBC= red blood cells, WBC = white blood cells. Hashed lines represented normal hematological value range for mice obtained from manufacturer. RBC data presented as million cells/microliter (M/µL), WBC and Platelets as thousand cells/uL (K/µL).

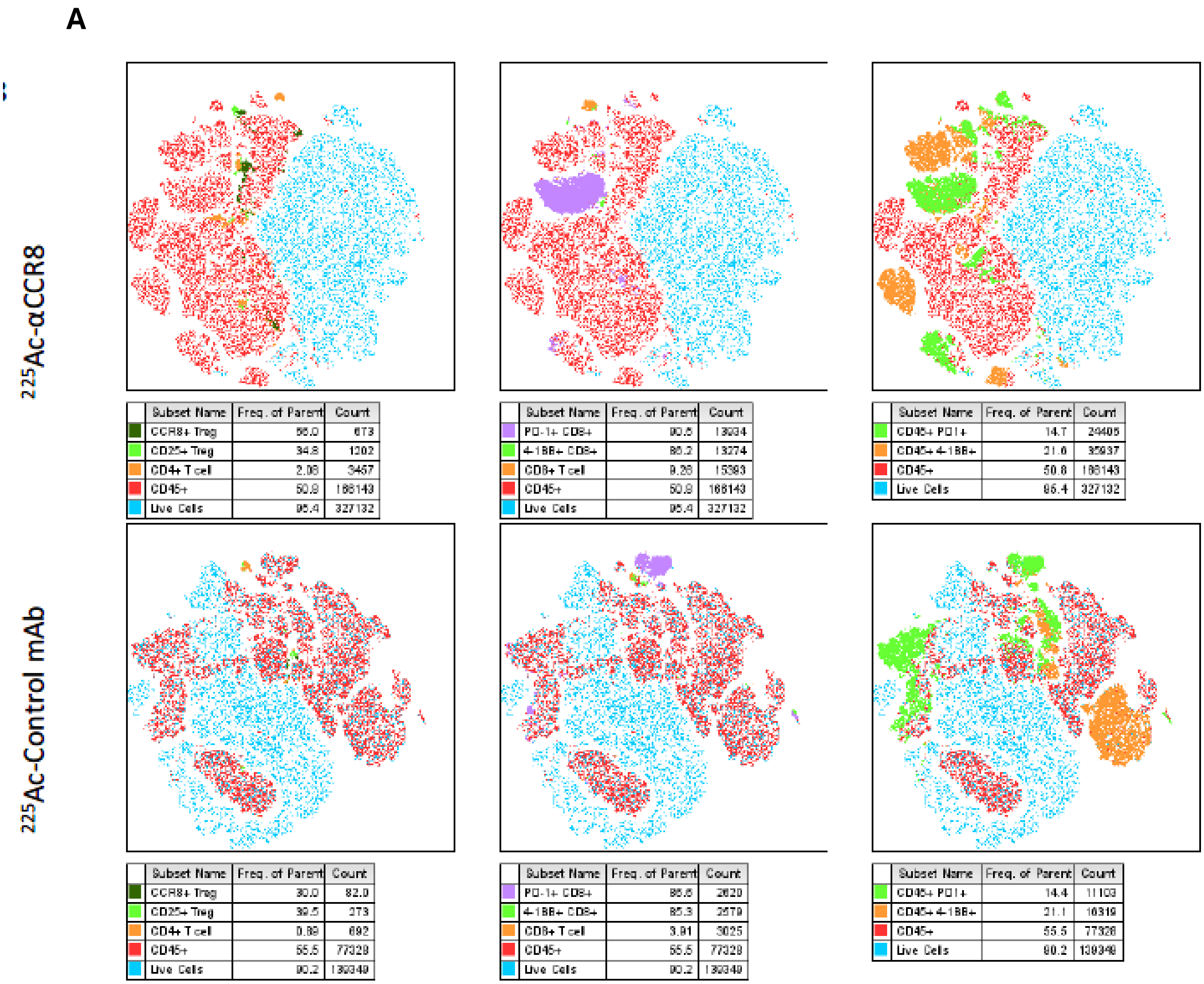

Supplementary Fig. S6A Representative t-SNE (t-distributed stochastic neighbor embedding) maps of tumor infiltrating CCR8+ Tregs, PD-1+CD8+ T cells and CD45+PD1+ T cells in MC38 tumor bearing mice treated with ^{225}Ac-anti-CCR8 or ^{225}Ac-control mAb on Day 3 (A) and Day 7 (B) post-treatment.

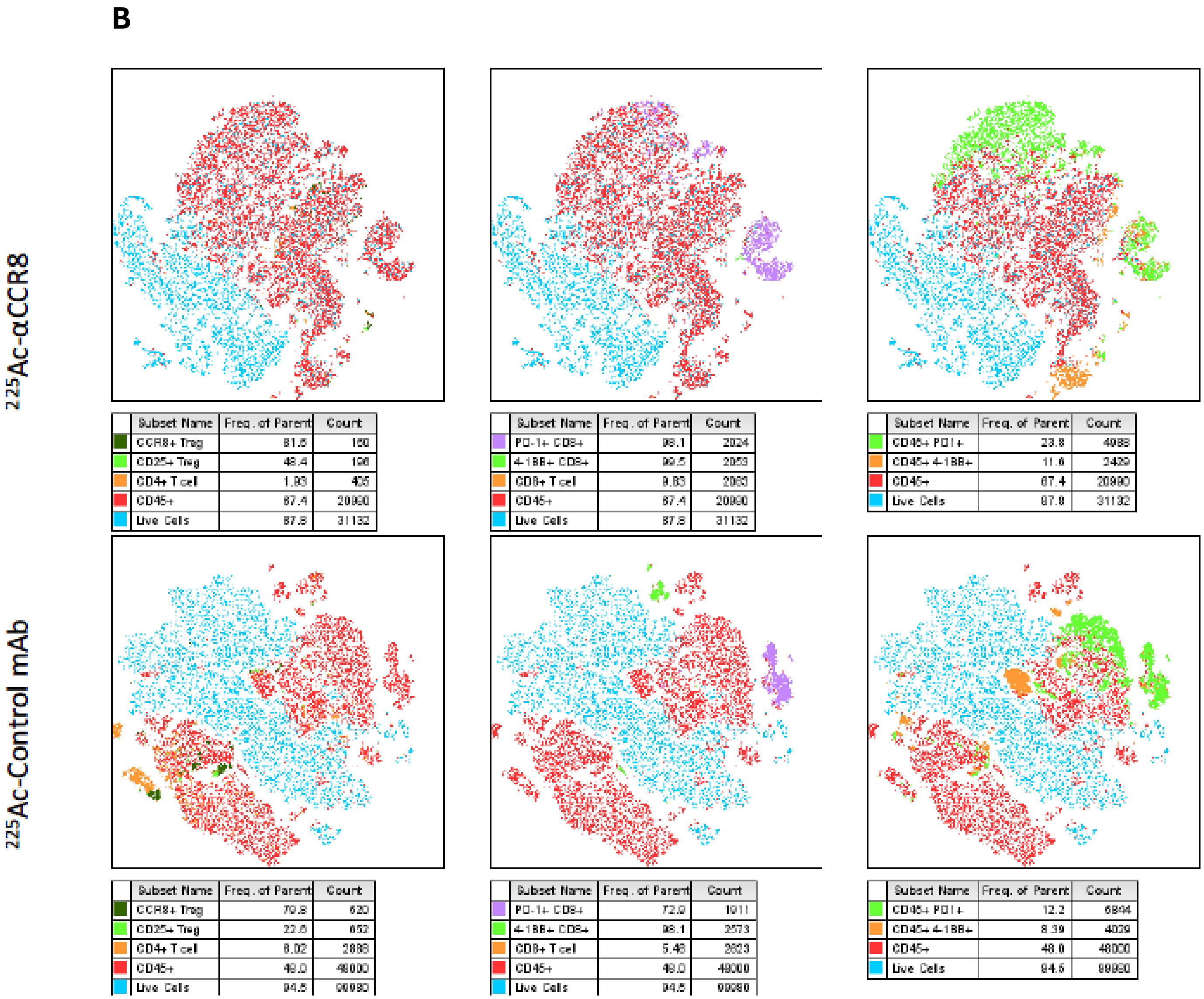

	Subset Name	Freq. of Parent	Count
	CCR8+ Treg	81.6	160
	CD25+ Treg	48.4	196
	CD4+ T cell	1.93	405
	CD45+	67.4	20980
	Live Cells	87.8	31132

	Subset Name	Freq. of Parent	Count
	PD-1+ CD8+	98.1	2024
	4-1BB+ CD8+	99.5	2053
	CD8+ T cell	9.83	2063
	CD45+	67.4	20980
	Live Cells	87.8	31132

	Subset Name	Freq. of Parent	Count
	CD45+ PD1+	23.8	4988
	CD45+ 4-1BB+	11.6	3429
	CD45+	67.4	20980
	Live Cells	87.8	31132

	Subset Name	Freq. of Parent	Count
	CCR8+ Treg	79.8	620
	CD25+ Treg	22.6	652
	CD4+ T cell	6.02	2888
	CD45+	48.0	48000
	Live Cells	94.5	99980

	Subset Name	Freq. of Parent	Count
	PD-1+ CD8+	72.9	1911
	4-1BB+ CD8+	98.1	2573
	CD8+ T cell	5.46	2623
	CD45+	48.0	48000
	Live Cells	94.5	99980

	Subset Name	Freq. of Parent	Count
	CD45+ PD1+	12.2	6844
	CD45+ 4-1BB+	8.39	4029
	CD45+	48.0	48000
	Live Cells	84.6	90980

Supplementary Fig. S6B Representative t-SNE (t-distributed stochastic neighbor embedding) maps of tumor infiltrating CCR8+ Tregs, PD-1+CD8+ T cells and CD45+PD1+ T cells in MC38 tumor bearing mice treated with ^{225}Ac-anti-CCR8 or ^{225}Ac-control mAb on Day 3 (A) and Day 7 (B) post-treatment.

A

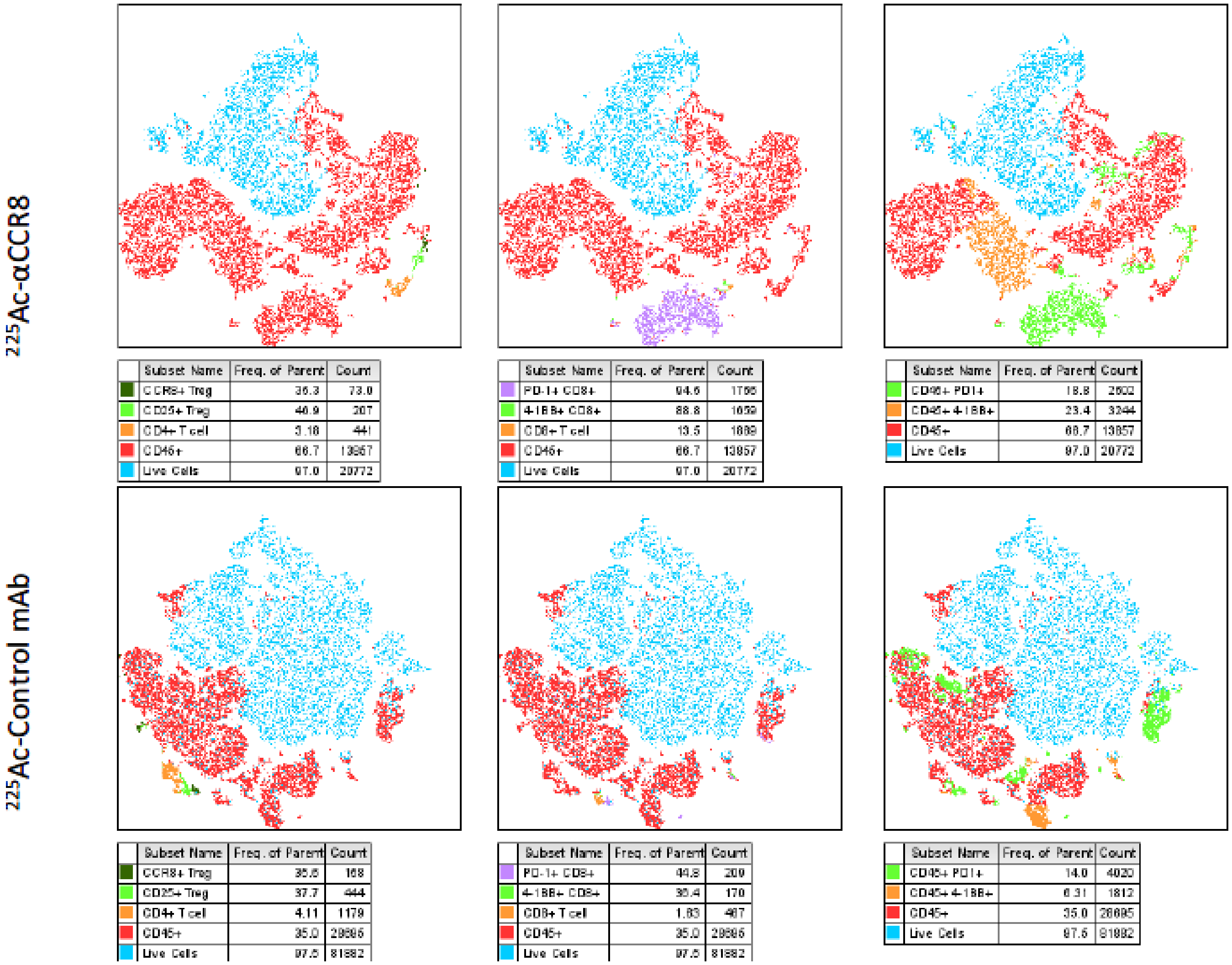

Supplementary Fig. S7A Representative t-SNE (t-distributed stochastic neighbor embedding) maps of tumor infiltrating CCR8+ Tregs, PD-1+CD8+ T cells and CD45+PD1+ T cells in CT26 tumor bearing mice treated with ^{225}Ac-anti-CCR8 or ^{225}Ac-control mAb on Day 3 (A) and Day 7 (B) post-treatment.

B

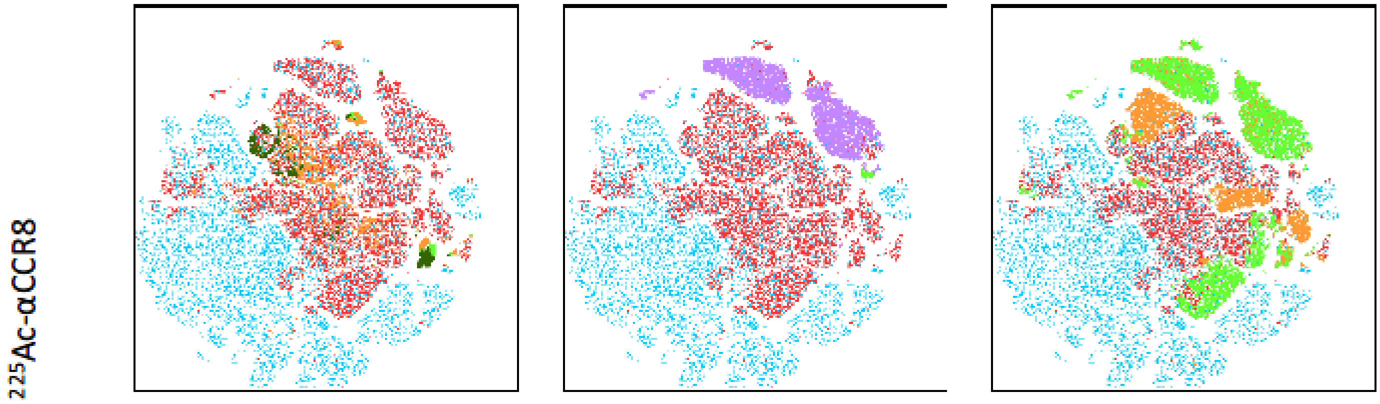

	Subset Name	Freq. of Parent	Count
	CCR8+ Treg	78.8	2465
	CD25+ Treg	42.7	3130
	CD4+ T cell	4.57	7327
	CD45+	52.4	160183
	Live Cells	93.2	306875

	Subset Name	Freq. of Parent	Count
	PD-1+ CD8+	98.0	25729
	4-1BB+ CD8+	99.9	26240
	CD8+ T cell	16.4	26264
	CD45+	52.4	160183
	Live Cells	93.2	305876

	Subset Name	Freq. of Parent	Count
	CD45+ PD1+	28.0	44881
	CD45+ 4-1BB+	24.8	39074
	CD45+	52.4	160183
	Live Cells	93.2	305875

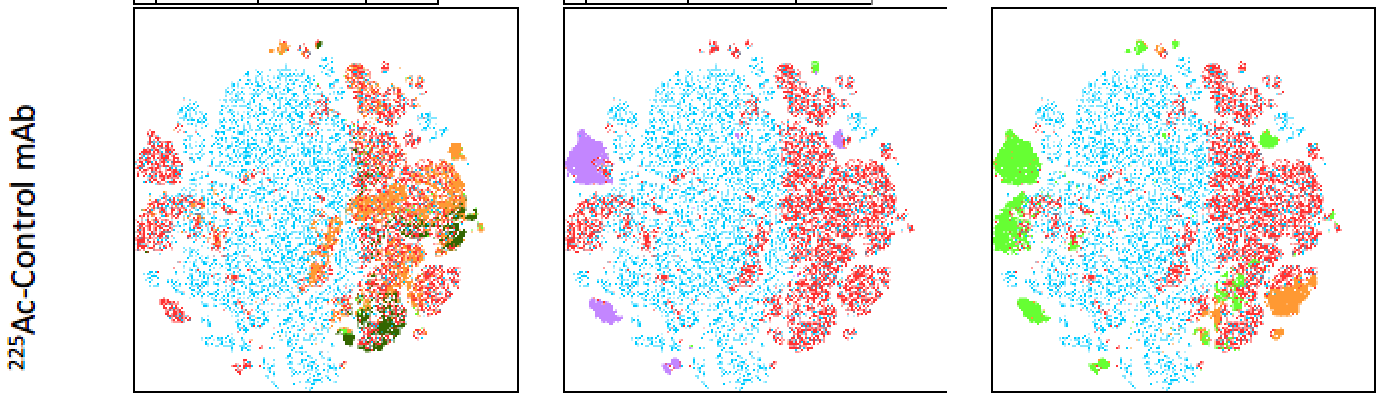

	Subset Name	Freq. of Parent	Count
	CCR8+ Treg	85.6	3499
	CD25+ Treg	29.9	4039
	CD4+ T cell	5.65	13492
	CD45+	39.7	238861
	Live Cells	95.0	6.02E5

	Subset Name	Freq. of Parent	Count
	PD-1+ CD8+	95.4	17593
	4-1BB+ CD8+	99.5	18346
	CD8+ T cell	7.72	18432
	CD45+	39.7	238861
	Live Cells	95.0	6.02E5

	Subset Name	Freq. of Parent	Count
	CD45+ PD1+	16.1	38362
	CD45+ 4-1BB+	10.3	24578
	CD45+	39.7	238861
	Live Cells	96.0	6.02E5

Supplementary Fig. S7B Representative t-SNE (t-distributed stochastic neighbor embedding) maps of tumor infiltrating CCR8+ Tregs, PD-1+CD8+ T cells and CD45+PD1+ T cells in CT26 tumor bearing mice treated with ^{225}Ac-anti-CCR8 or ^{225}Ac-control mAb on Day 3 (A) and Day 7 (B) post-treatment.

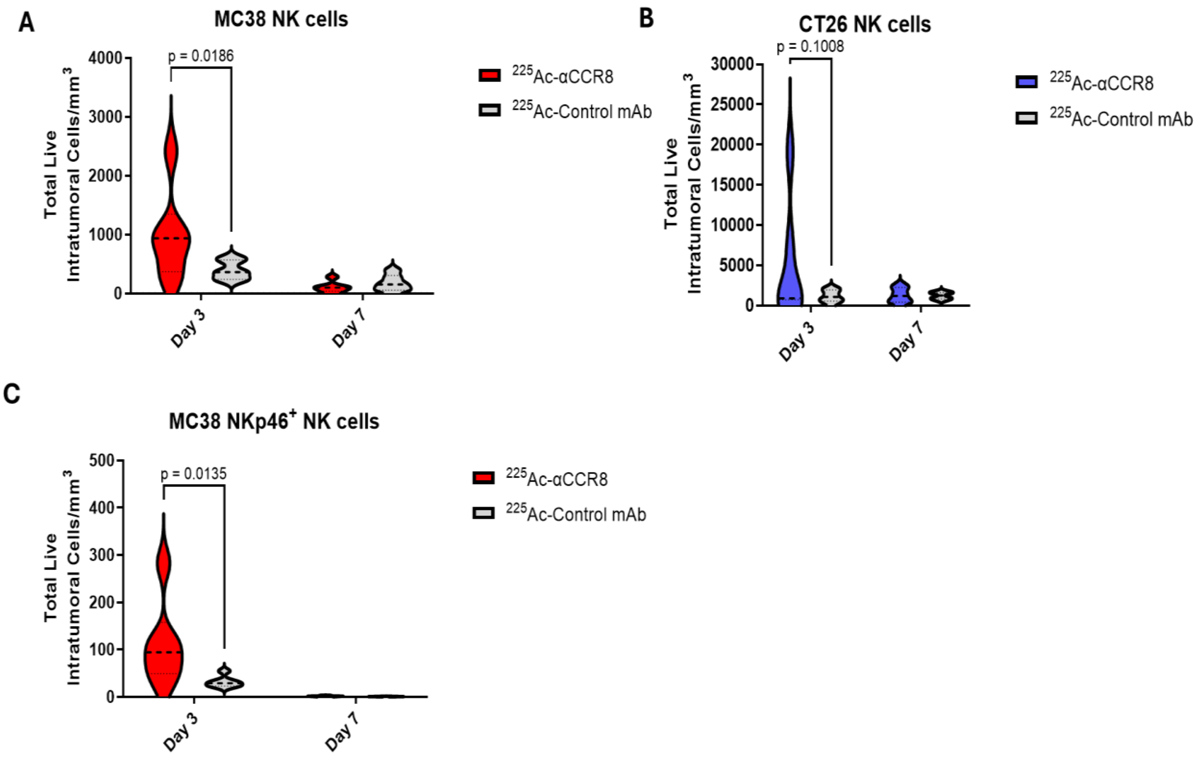

Supplementary Figure S8. ^{225}Ac-anti-CCR8 RIT expands initial anti-tumor NK cells by Day 3 post administration. A) NK cells in MC38 tumors, B) NK cells in CT26 tumors, C) NKp46+ NK cells in MC38 tumors.
